# Uncovering a novel biosynthetic gene cluster for sordarin through genome mining in the fungus *Talaromyces adpressus*

**DOI:** 10.1186/s40643-025-00864-x

**Published:** 2025-04-17

**Authors:** Qianqian Xu, Xiaomeng Ren, Linzhen Hu, Qiaoxin Xu, Xiaodong Zhang, Mengyi Deng, Ying Ye, Yonghui Zhang, Yuanyuan Lu, Yuben Qiao

**Affiliations:** 1 Maternal and Child Health Hospital of Hubei Province, Tongji Medical College, Huazhong University of Science and Technology, Wuhan, 430033 People’s Republic of China; 2 State Key Laboratory of Biocatalysis and Enzyme Engineering, School of Life Sciences, Hubei University, Wuhan, 430062 People’s Republic of China; 3 Hubei Key Laboratory of Natural Medicinal Chemistry and Resource Evaluation, School of Pharmacy, Tongji Medical College, Huazhong University of Science and Technology, Wuhan, 430030 People’s Republic of China; 4 Innovation Laboratory for Sciences and Technologies of Energy Materials of Fujian Province (IKKEM), Xiamen, 361005 People’s Republic of China; 5 School of Life Science and Engineering, Southwest Jiaotong University, Chengdu, Sichuan 610031 People’s Republic of China

**Keywords:** Sordarin, Biosynthetic gene cluster, *Talaromyces adpressus*, New compound, Anti-tumor activities

## Abstract

To explore the chemical and biological diversities of diterpenoids from the fungus *Talaromyces adpressus*, a previously unknown biosynthetic gene cluster (BGC, *tdn*) for sordarin (a well-known fungal antibiotics) was discovered by leveraging the genome mining method. Heterologous expressions of key genes of *tdn* in *Aspergillus oryzae*, led to the determination of one new diterpenoid, cycloaraneosene-9-ol-8-one (**4**), and three known diterpenoids, cycloaraneosene (**1**), cycloaraneosene-9-ol (**2**), cycloaraneosene-8,9-diol (**3**). The structures of **1**–**4** was elucidated well via detailed analysis of 1D and 2D NMR, GCMS, HRESIMS, IR data, and comparison with reported data. Structurally, compounds **1**–**4** were belonging to fusicoccane diterpenoids with a classical tricyclic 5/8/5 ring system, which are participated in the biosynthesis of sordarin. Compound **4** maybe a key precursor for a Baeyer–Villiger like reaction with C8–C9 bond cleavage in the biosynthetic pathway of sordarin. Moreover, all isolates were evaluated for their bioactivities, compounds **3**, and **4** exhibited inhibitory activities against the human cancer cell lines with IC_50_ values ranging from 7.8 to 32.4 *µ*M. **3** and **4** promote cell apoptosis of HCT-116 and HepG2 cells, and suppress cell migration of HepG2 cells. As well, **3** and **4** also decrease gene expression of cell proliferation related molecules BCL-2 and cyclin D1, while increase expression of cell apoptosis related gene BAX. Targets predication and molecular docking indicate that compound **4** exhibits stronger affinity for DBL, suggesting its excellent binding potential. This finding will be enriched the structures and bioactivities of diterpenoids with a tricyclic 5/8/5 ring system, most importantly, will provide new strategies for the synthetic biological research of sordarins.

**Graphical Abstract:**

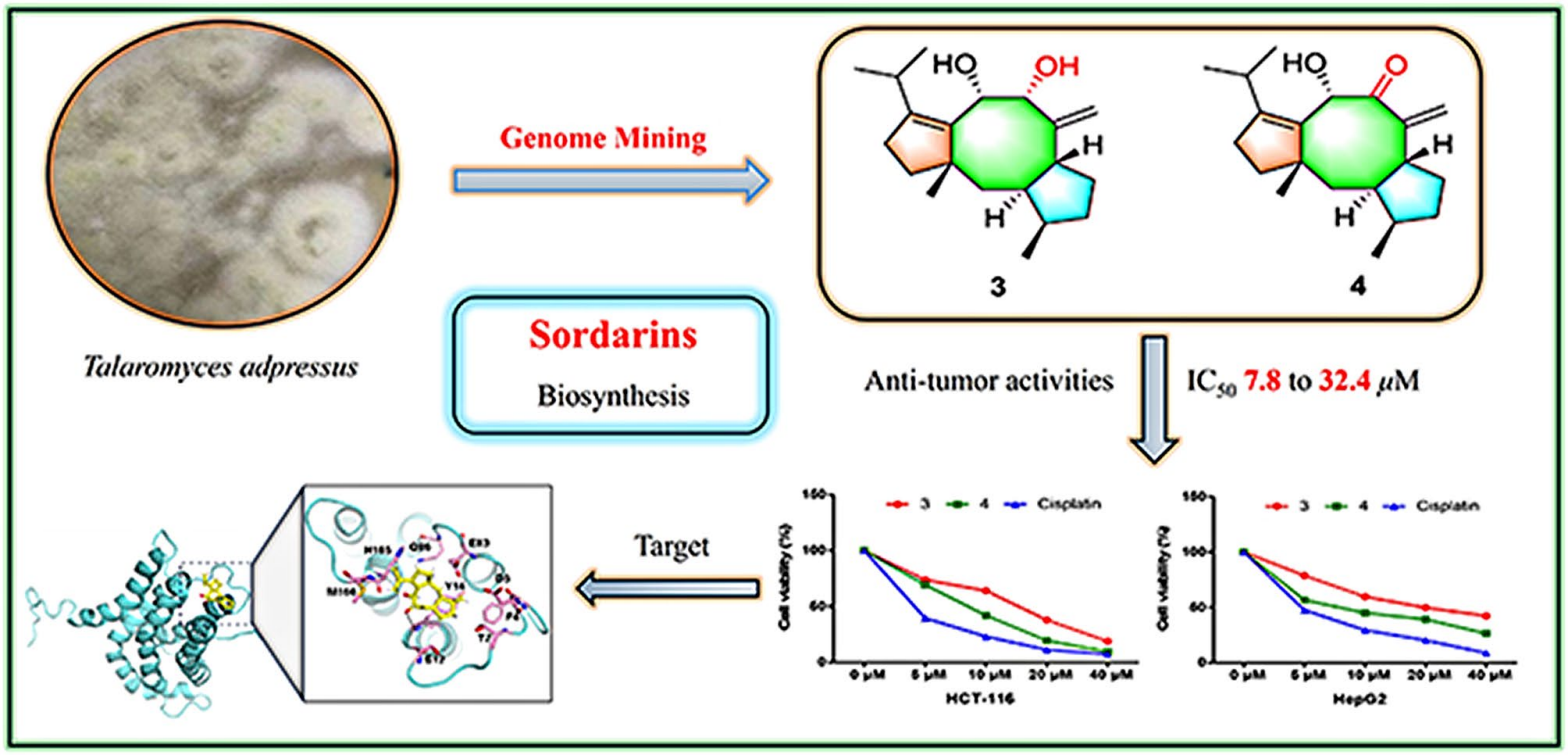

**Supplementary Information:**

The online version contains supplementary material available at 10.1186/s40643-025-00864-x.

## Introduction

**Scheme 1:**
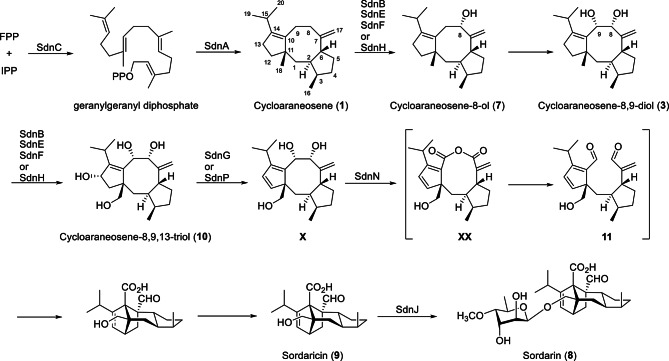
Proposed biosynthetic pathway for sordarin

Sordarin (**8**, CAS 11076-17-8, Scheme 1) is a diterpene glycoside with anti-fungal bioactivities (Justice et al. 1998; Shao et al. 2022 et al., 2007) which was first isolated from the fungus *Sordaria araneosa* Cain (Hauser and Sigg 1971). To date, lots of natural analogues of **8** were discovered from diverse fungi, such as GR135402 and BE31405 with differ modifications (Fostel and Lartey 2000; Kinsman et al. 1998; Nonaka et al. 2015). Structurally, sordarin is a highly complex natural diterpenoid with a unique 5/6/6 tricyclic system featuring a bicyclo[2.2.1]heptane norbornene-like framework. This special construction derived from a C8–C9 bond cleavage and a rearrangement of the fusicoccane diterpenoids with a classical tricyclic 5/8/5 ring system carbon skeleton, which is exhibiting diverse bioactivities (Hu et al. 2018; Li et al. 2019, 2024; Liu and Hong 2024; Tang et al. 2016).

Pharmacologically, sordarin can inhibit protein synthesis in fungi by stabilizing the ribosome eEF2 complex. It catalyzes the translocation of tRNA and mRNA after the formation of peptide bonds during translation (Fostel and Lartey 2000; Shao et al. 2022). Due to the unique structural and drug potential, sordarins have attracted greatest attention from the chemists (Büschleb et al. 2016; Chiba et al. 2006; Liang 2008; Liang et al. 2007; Mander and Thomson 2003, 2005; Schulé et al. 2009). However, these syntheses suffered from lengthy steps and low overall yield. Therefore, precise elucidation of biosynthetic pathways, especially discover the enzyme(s) catalyzing C8–C9 bond cleavage and all the intermediates in their biosynthetic pathway, will provide new strategies for synthetic biological development of sordarins.

**Fig. 1:**
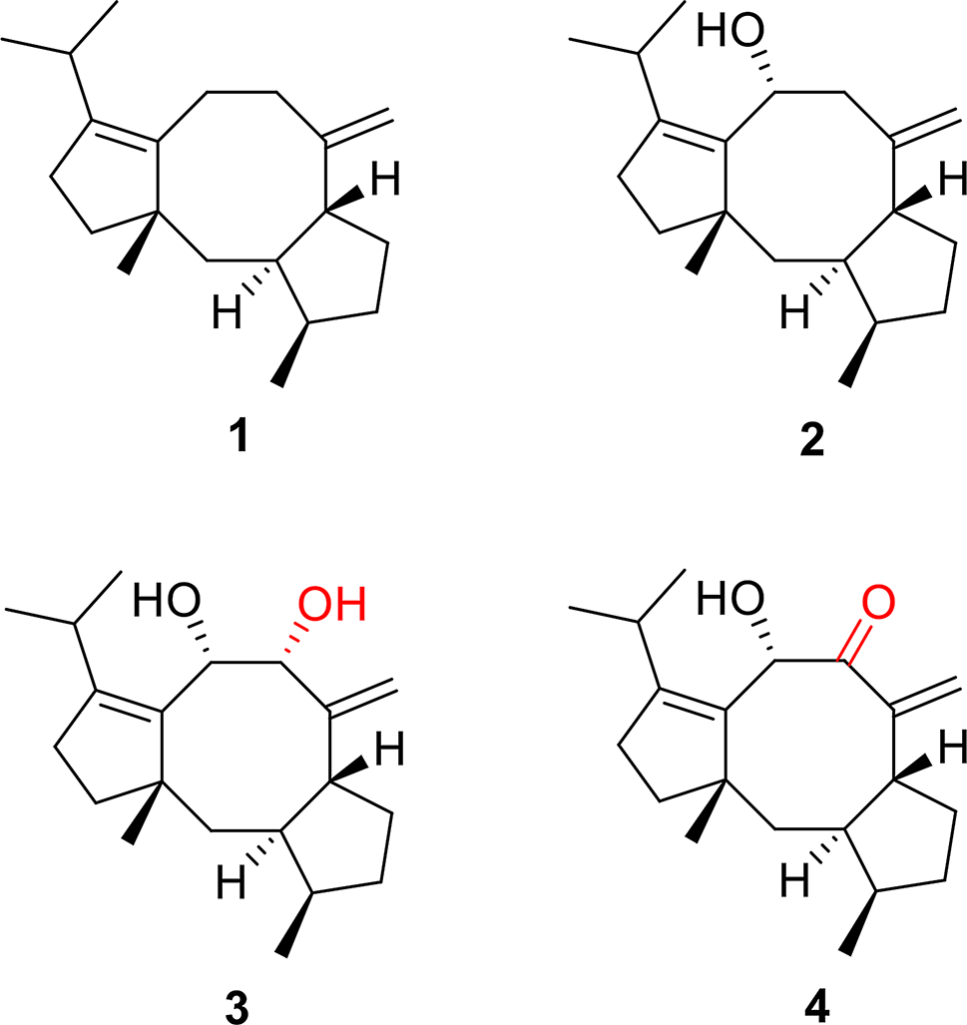
Chemical structures of compounds **1**–**4**

**Fig. 2:**
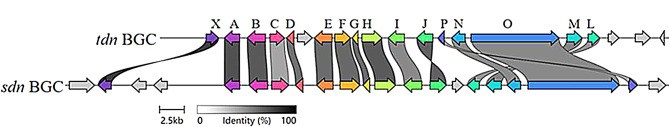
The BGCs of sordarin. *Tdn* from *T. adpressus* and *sdn* from *S. araneosa* Cain ATCC 36386

**Fig. 3:**
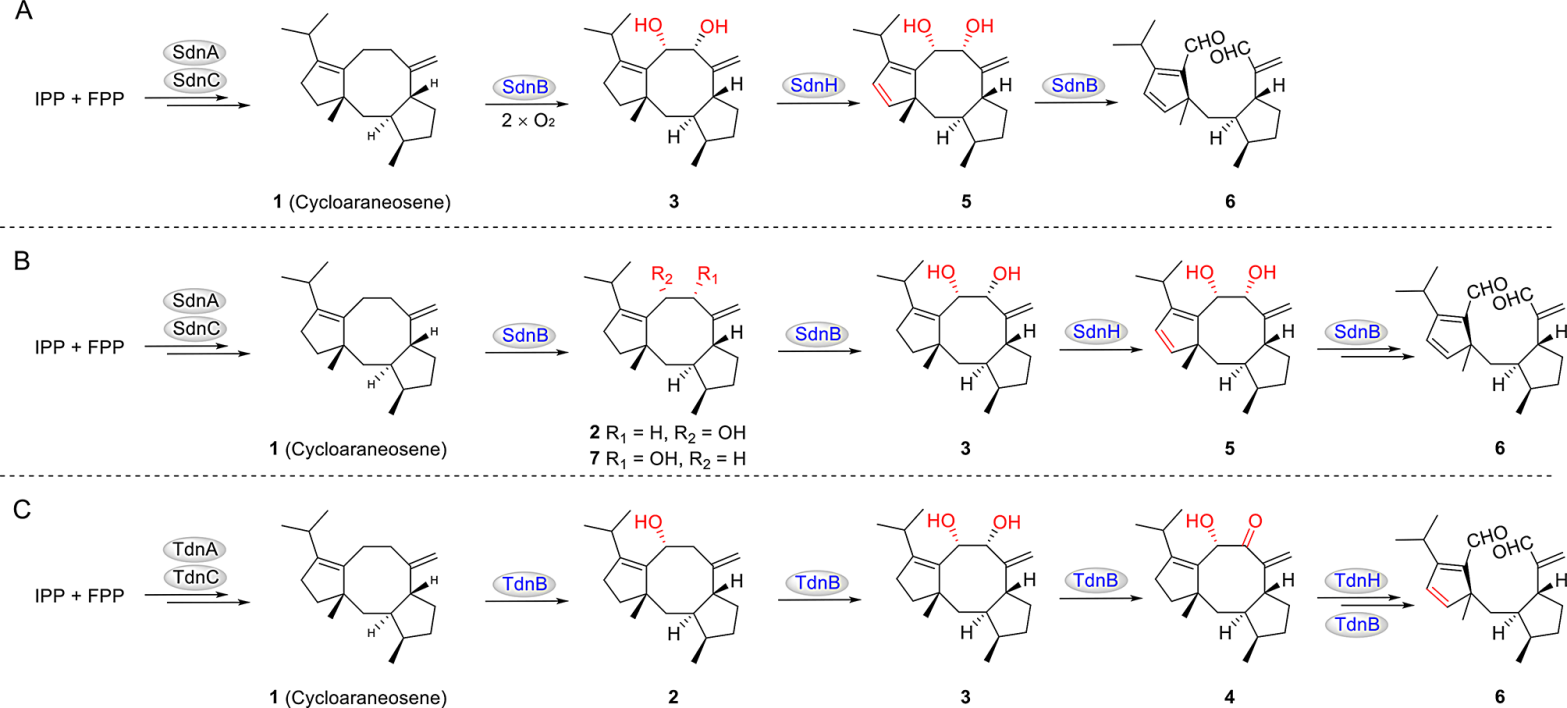
Proposed biosynthetic pathway of sordarin intermediates. (**A**) Tang reported the key intermediate is a vicinal dihydroxy **3**; (**B**) Ge reported pathway; (**C**) We deduced pathway showing **4** maybe a key intermediate

According to an early biochemical experiment focused on the BGC (*sdn*, Fig. 2) for sordarin synthesis from the genome of *Sordaria araneosa* Cain ATCC 36386 (Kudo et al. 2016). SdnAC are responsible for the formation of cycloaraneosene (**1**, Scheme 1). Four cytochrome P450 monooxygenases (SdnB/E/F/H) catalyze multiple steps of oxygenation reactions. In turn, a FAD-dependent oxygenase (SdnN) catalyzes **X** to yield **XX** with Baeyer–Villiger like reaction. Recently, two groups published biosynthetic research for sordarin (Liu et al. 2022; Sun et al. 2022). A GGPP synthase (SdnC) and a diterpene cyclase (SdnA) generates the 5/8/5 cycloaraneosene structure, which is modified by a set of P450s (SdnBEFH) that catalyze a series of reactions, involving in hydroxylation, desaturation, and C − C bond oxidative cleavage, to give a carboxylate intermediate with a terminal alkene and a cyclopentadiene moiety (Scheme 1 and Fig. 3). In turn, a novel Diels–Alderase SdnG catalyzes an intramolecular Diels–Alder reaction with clear mechanism on this intermediate to form core structure of sordarin (Sun et al. 2025). And they believed that the key intermediate is a vicinal dihydroxy compound **3** (Figs. 3 A and **3**B). However, all of them do not get the precursor **4** (Fig. 1) for B–V product **XX**, which maybe a key intermediate in the biosynthetic pathway according to the previous propose (Scheme 1).

On our program continuing to phytochemically investigate novel natural products from the *Talaromyces* species, and elucidate their biosynthesis (Chaudhary et al. 2020; Li et al. 2023; Liu et al. 2024; Meijia et al. 2024; Wang et al. 2024; Zhang et al. 2020, 2021, 2022; Zheng et al. 2023, 2024a, b), we have discovered a previously unknown BGC (*tdn*) for sordarin by leveraging the genome mining in *T. adpressus*. The fungus *T. adpressus* stands out due to its unique characteristics and potential, making it an ideal candidate for phytochemical investigations. A detailed bioinformatic analysis and protein blast (Yuan et al. 2024) showed that *tdn* cluster has high homology with the *sdn* gene cluster (Fig. 2; Table 1). Through heterologous expression of *tdn* genes in *A. oryzae*, we identified one new compound **4**, which maybe a key intermediate in the biosynthetic pathway of sordarins, and three known precursors **1**–**3** (Fig. 1).

Here, we report the BGC identification, structural elucidation and the biological activity of all the isolates, which will be improving the deeply chemical, biosynthetic, and pharmacological studies for sordarins. The newly identified gene cluster holds immense promise and significance, not only advancing our understanding of sordarin’s biosynthesis but also highlighting its crucial role in the broader field of biology.

**Table 1:** *Tdn* biosynthetic gene designation and their putative functions, and the protein identity between *Sdn* and *tdn*

Sdn	Tdn	Putative function	%Identity
*sdnA*	*tdnA*	Cycloalaneosene synthase	78.49%
*sdnB*	*tdnB*	Cytochrome P450 monooxygenase	74.63%
*sdnC*	*tdnC*	GGPP synthase	62.95%
*sdnD*	*tdnD*	Sugar *O*-methyltransferase	70.45%
*sdnE*	*tdnE*	Cytochrome P450 monooxygenase	73.80%
*sdnF*	*tdnF*	Cytochrome P450 monooxygenase	73.74%
*sdnG*	*tdnG*	Diels–Alderase	70.55%
*sdnH*	*tdnH*	Cytochrome P450 monooxygenase	70.83%
*sdnI*	*tdnI*	GDP-D-mannose-4,6-dehydratase	68.03%
*sdnJ*	*tdnJ*	GDP-6-deoxy-D-altrose	73.41%
*sdnL*	*tdnL*	PKS methyltransferase	56.82%
*sdnM*	*tdnM*	Transcriptional regulator	61.94%
*sdnN*	*tdnN*	FAD-dependent oxygenase	61.12%
*sdnO*	*tdnO*	Polyketide synthase	61.27%
*sdnP*	*tdnP*	Hypothetical protein	68.56%
*sdnX*	*tdnX*	Fe(II)/αKG-dependent dioxygenase	83.56%

## Materials and methods

### General

Optical rotations, UV, and FT-IR data were recorded on a PerkinElmer 341 instrument, a Varian Cary 50 instrument, and a Bruker Vertex 70 instrument with KBr pellets, respectively. CD data were measured with a JASCO-810 CD spectrometer instrument. The high-resolution electrospray ionization mass spectra (HRESIMS) were recorded by using a positive ion mode on a Thermo Fisher LC-LTQ-Orbitrap XL instrument. One- and two-dimensional NMR data were recorded on a Bruker AM-400 instrument, with the reference of ^1^H and ^13^C NMR chemical shifts of the solvent peaks for CH_3_Cl-*d* (*δ*_H_ 7.26 and *δ*_C_ 77.0). Column chromatography (CC) was carried out by using silica gel (80 − 120, 100 − 200, 200 − 300 mesh, Qingdao Marine Chemical, Inc., Qingdao, People’s Republic of China), Lichroprep RP-C_18_ gel (40–63 *µ*m, Merck, Darmstadt, Germany), and Sephadex LH-20 (GE Healthcare Bio-Sciences AB, Sweden).

### Strains

The fungus *T. adpressus* was isolated from the soil collected from Yalong Bay in Sanya, Hainan. Its sequence data has been submitted to DDBJ/EMBL/GenBank under accession No. MZ373307. And the *tdn* gene cluster sequence data can be found in GenBank under accession No. PQ878474. A voucher sample is preserved in the China Center for Type Culture Collection (CCTCC M 20232471), Wuhan University.

*Escherichia coli* DH5*α* was used for cloning and following standard recombinant DNA techniques. A fungal host strain used in this study was *A. oryzae* NSAR1, a quadruple auxotrophic mutant (*niaD*^*-*^, *sC*^*-*^, *argB*^*-*^, *adeA*^*-*^) for fungal expression (Tazawa et al. 2018; Ye et al. 2015). *T. adpressus* was used for genomic DNA extraction, its genome sequence was used to design the PCR primers.

### Preparation of expression plasmids

The *tdnA*, *tdnB tdnC*, *tdnE*, *tdnF*, and *tdnH* were amplified from the genomic DNA of *T. adpressus* with the primer set shown in Table S1. Linker region in the designed plasmids were amplified from pAdeA2 by PCR with KOD-Fx-Neo (TOYOBO). Three expression plasmids were constructed as follows. Gene *tdnA* and *tdnC* was inserted into the NheI site and KpnI of pAdeA2 using an NEBuilder^®^ HiFi DNA Assembly Cloning Kit (NEBuilder HiFi DNA Assembly Master Mix, New England BioLabs) to construct pAdeA2-*tdnAC*. The KpnI- and NheI-digested fragment of pUARA2 plasmid was subjected to Gibson assembly (NEBuilder HiFi DNA Assembly Master Mix, New England BioLabs) with *tdnB*, *tdnE*, *tdnF*, and *tdnH*.

### Transformation of A. oryzae

Transformation of *A. oryzae* was performed by the protoplast-polyethylene glycol method reported previously to construct the two transformants (AO-tdnAC and AO-tdnACBEFH) (Qiao et al. 2022). Plasmids used for the construction of each transformant are summarized in Table S2.

### Fermentation, extraction, and purification

Transformants were cultured in MPY liquid culture medium (3% maltose, 1% hipolypeptone, 0.5% yeast extract, 10 mg adenine, 100 mL) in 500 mL Erlenmeyer flasks incubated at 30 ˚C for 3 days, each containing 200 mL of MPY medium, with additional nutrients lacking in the transformed plasmid. Pre-sterilize by autoclaving, and culture for 2–5 L at 30 ℃ and 220 rpm for 1 week.

The liquid medium was extracted five times with ethyl acetate, and the solvent was removed by concentration using a rotary evaporator to obtain a total product. The culture product of the transformant was first filtered through a small amount of positive phase silica gel to remove insoluble impurities. The total product of AO-TdnAC was purified by normal phase silica gel chromatography column with petroleum ether elution to obtain the skeleton compound **1**. The AO-TdnACBEFH cultured product was eluted with a normal phase silica gel column using petroleum ether-ethyl acetate to yield compounds **2**–**4**.

### Spectroscopic data

Compound **3**: colorless oil; ^1^H NMR (600 MHz, CDCl_3_) data: *δ*_H_: 5.16 (1H, brs, H-17a), 4.95 (1H, brs, H-17b), 4.50 (1H, dt, *J* = 5.2, 2.2 Hz, H-8), 4.37 (1H, d, *J* = 5.2 Hz, H-9), 3.16 (1H, sept, *J* = 6.8 Hz, H-15), 2.17 (1H, m, H-13a), 2.13 (1H, m, H-5a), 2.03 (1H, m, H-3), 2.02 (1H, m, H-13b), 1.89 (1H, m, H-6), 1.40 (1H, m, H-3), 1.83 (1H, m, H-5b), 1.68 (1H, m, H-1a), 1.66 (1H, m, H-2), 1.59 (1H, m, H-4a), 1.55 (1H, m, H-12a), 1.38 (1H, m, H-4b), 1.34 (1H, m, H-1b), 1.32 (1H, m, H-12b), 0.98 (3 H, s, H_3_-18), 0.97 (3 H, d, *J* = 6.7 Hz, H_3_-19), 0.94 (3 H, d, *J* = 7.0 Hz, H_3_-20), 0.79 (3 H, d, *J* = 7.1 Hz, H_3_-16). ^13^C NMR (150 MHz, CDCl_3_) data: *δ*_C_: 155.7 (C-7), 146.8 (C-14), 137.9 (C-10), 106.4 (C-17), 76.2 (C-8), 69.1 (C-9), 50.5 (C-11), 50.3 (C-2), 45.5 (C-6), 42.9 (C-12), 41.4 (C-3), 34.5 (C-5), 34.3 (C-1/4), 28.3 (C-15), 27.2 (C-18), 26.7 (C-13), 21.7 (C-20), 20.6 (C-19), 14.6 (C-16).

Compound **4**: colorless oil; ^1^H NMR (600 MHz, CDCl_3_) data: *δ*_H_: 5.87 (1H, d, *J* = 0.6 Hz, H-17a), 5.38 (1H, d, *J* = 1.0 Hz, H-17b), 4.83 (1H, d, *J* = 2.2 Hz, H-9), 2.52 (1H, sept, *J* = 6.8 Hz, H-15), 2.35 (1H, m, H-6), 2.22 (1H, m, H-13a), 2.17 (1H, m, H-3), 2.11 (1H, m, H-13b), 2.10 (1H, m, H-5a), 1.79 (1H, m, H-4a), 1.77 (1H, m, H-5b), 1.73 (1H, m, H-12a), 1.71 (1H, m, H-2), 1.62 (1H, m, H-1a), 1.51 (1H, m, H-12b), 1.50 (1H, m, H-4b), 1.45 (1H, m, H-1b), 1.12 (3 H, s, H_3_-18), 0.91 (3 H, d, *J* = 6.8 Hz, H_3_-20), 0.89 (3 H, d, *J* = 7.1 Hz, H_3_-16), 0.77 (3 H, d, *J* = 6.8 Hz, H_3_-19). ^13^C NMR (150 MHz, CDCl_3_) data: *δ*_C_: 203.5 (C-8), 153.9 (C-7), 150.8 (C-14), 134.4 (C-10), 120.0 (C-17), 70.0 (C-9), 50.3 (C-11), 49.5 (C-2), 45.7 (C-6), 44.1 (C-12), 41.5 (C-3), 34.7 (C-5), 34.6 (C-1), 32.6 (C-5), 27.2 (C-13), 27.0 (C-15), 26.2 (C-18), 21.0 (C-20), 19.2 (C-19), 14.9 (C-16).

### Cell culture, administration and detection

Human cancer cells were purchased from Procell Life Science & Technology Co., Ltd (Wuhan, China). Cells were cultured in DMEM (Hyclone, USA) supplemented with 10% fetal bovine serum (Procell, China) and 1% antibiotics of penicillin/streptomycin (100 units/mL) (Invitrogen, USA). Cells were grown under an atmosphere of 5% CO_2_ in air at 37 °C. For the cell viability assay, cells were incubated with 5, 10, 20 and 40 µM of tested compounds for 48 h, with cisplatin as a positive control. Then cell counting kit 8 (CCK-8, Dojindo Laboratories, Japan) was added to each well for 2 h, and detected of absorbance at 450 nm. The data are expressed as averages of three replicates. The IC50 values were calculated by using a standard dose–response curve fitting with Prism (version 5.0, GraphPad Software, La Jolla, CA, USA). For the cell apoptosis, cells were incubated with 5, 10, 20 and 40 µM of **3** or **4** for 6 h. Then the cells were determined using an Annexin V-fluorescein isothiocyanate (FITC) apoptosis detection kit (KeyGEN, Jiangsu, China) according to the manufacturer’s protocol.

### Quantitative real time PCR (qRT-PCR) tests

Total RNA was reverse-transcribed into cDNA utilizing a transcription kit (Vazyme Biotech Co.,Ltd, Nanjing, China). qRT-PCR was performed using SYBR Green qPCR Mix (Vazyme Biotech Co.,Ltd, Nanjing, China). This reaction contained 0.2 µM of both forward and reverse primers in a final reaction volume of 10 µL and was detected by ABI QuantStudio 5 (Thermo Fisher Scientific, USA). The amplification of the resulting cDNA involved an initial incubation at 95 °C for 5 min, followed by 40 cycles of denaturation at 95 °C for 10 s, annealing at 60 °C for 20 s, and extension at 72 °C for 30 s. The expression values were normalized relative to β-actin. The corresponding primer sequences were listed in Supplementary Table S3.

### Transwell assay

HepG2 cells were incubated with compound **3** and **4** for a duration of 48 h. Subsequently, a suspension of 5 × 10^4^ cells was prepared in serum-free DMEM, and these cells were introduced into inserts (8 μm, BD Biosciences, USA) The chambers were then supplemented with DMEM containing 20% fetal bovine serum. Following an additional 8-hour incubation, the upper membrane was stained using crystal violet. Representative microscopic fields were carefully selected and examined under an Olympus fluorescence microscope (Tokyo, Japan).

### Molecular docking

The initial DBL homology structure (Table S4) was obtained from the Protein Data Bank (PDB ID: 1BY1) (Aghazadeh et al. 1998). Molecular docking simulations were carried out using YASARA v20.10.4. Missing hydrogen atoms in the DBL structure were added the LEAP module of Amber 18, and the residues were optimized with the AMBER14 force field to accurately represent protein interactions (Case et al. 2023). To neutralize the overall charge of the system, 18 Na + ions were added to the protein surface. Ligand compounds were parameterized using the General AMBER Force Field (GAFF), with partial atomic charges and missing parameters derived from the RESP method at the HF/6-31G* theory level (Bayly et al. 1993; Wang et al. 2004). A cubic simulation cell was generated, extending 5 Å around all solute atoms to allow sufficient space for ligand movement and interactions. Docking runs were performed, and the binding modes of the ligand were evaluated to identify critical interactions with key residues. Visualization and detailed inspection of the docking poses were conducted using PyMOL 2.5, allowing the identification of significant interactions between the ligand and active site residues, and providing insights into the binding mechanism and reactivity.

### Statistical and analysis

Gene cluster comparison of *tdn* and *sdn* was conducted in clinker (https://github.com/gamcil/clinker); gene designation using 2ndFind (https://biosyn.nih.go.jp/2ndfind/); protein blast on NCBI (https://blast.ncbi.nlm.nih.gov/Blast.cgi) and uniprot (https://www.uniprot.org/align). The chemical structure and Canonical SMILES of compounds **3** and **4** were imported into the PharmMapper server (https://www.lilab-ecust.cn/pharmmapper/) to identify potential targets. All experiments were conducted in biological triplicate. All data are displayed as the means ± standard error of the mean (SEM). Differences were evaluated by Student’s t-test, and *P < 0.05 was considered statistically significant.

## Results and discussion

### Heterologous expression in A. oryzae

**Fig. 4:**
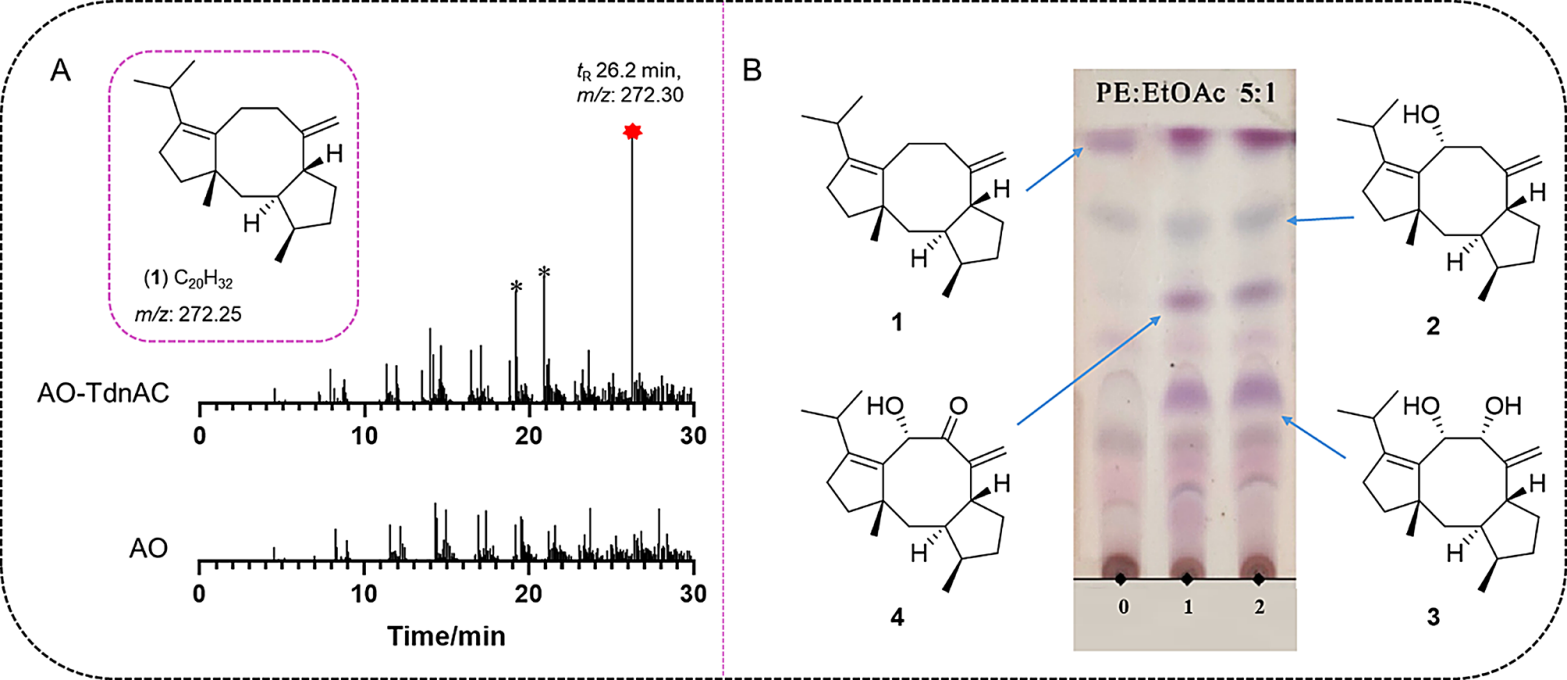
Heterologously expressing functional genes in AO. (**A**) Expressing *tdnAC*; (**B**) Expressing *tdnACBEFH*. “0” represents transformant AO-tdnAC, while “1” and “2” represent transformants AO-tdnACBEFH-1# and AO-tdnACBEFH-2#; PE (petroleum ether), EtOAc (ethyl acetate). *Indicates the compounds which are not relevant to **1**–**4**

Initially, we using SdnA protein sequence as the probe to search homologous protein in the genome of *T. adpressus*, which led to the identification of the *tdn* gene cluster. To investigate the products of *tdn* cluster, we heterologously expressed the functional genes in *A. oryzae* NSAR1 (Qiao et al. 2022; Ye et al. 2015). The result of heterologously expressing *tdnAC* exhibited new peak with *m/z* 272.30 [M]^+^ at *t*_R_ 26.2 min in metabolites of AO-tdnAC (Fig. 4A), compared with the control AO-NSAR1. The product appears purple on the thin layer chromato-graphy (TLC) using vanillin analysis (Fig. 4B). Then, the transformant AO-tdnACBEFH containing four P450 enzymes was constructed and expressed according to previous reports. This work led to the obtain three new points from its metabolites based on TLC experiment (Fig. 4B). Subsequently, a large-scale fermentation of the positive strain and TLC guided isolation were applied to obtain colorless oil compounds **1**–**4** (Fig. 1).

### Structure elucidation

Compounds **1**–**4** were isolated as colorless oil; On the basis of detailed NMR data analysis of **1**–**3** (Figs. S1, S2, and S5–11), we elucidated their structure as cycloaraneosene (**1**) and cycloaraneosene-9-ol (**2**) (Kudo et al. 2016), cycloaraneosene-8,9-diol (**3**) (Sun et al. 2022). It is worth mentioning that compound **3** is a new compound published during our research (Kudo et al. 2016; Sun et al. 2022). Therefore, we also present the experimental data of **3** in the article (Table 2).

The molecular formula C_20_H_30_O_2_ of new compound **4** was determined via HRESIMS (Fig. S3B) at *m/z* 325.2138 [M + Na]^+^ (calcd for C_20_H_30_O_2_Na, 325.2143), referring to six degrees of unsaturation. The IR spectrum (Fig. S4C) showed absorption bands at 2959, 1688, and 1651/1613 cm^− 1^, indicating the existence of hydroxyl, carbonyl, and alkene groups in **4**. The 1D ^1^H NMR, ^13^C NMR, and DEPT, and 2D HSQC spectroscopic data (Table 2) of **4** revealed 20 carbon resonances that were attributed to four methyls: CH_3_-18 (*δ*_H_ 1.12, s, *δ*_C_ 26.2), CH_3_-20 (*δ*_H_ 0.91, d, *J* = 6.8 Hz, *δ*_C_ 21.0), CH_3_-16 (*δ*_H_ 0.89, d, *J* = 7.1 Hz, *δ*_C_ 14.9), CH_3_-19 (*δ*_H_ 0.77, d, *J* = 6.8 Hz, *δ*_C_ 19.2), and; six methylenes including one olefined: CH_2_-17 (*δ*_H_ 5.87, 5.38, *δ*_C_ 120.0), CH_2_-12 (*δ*_H_ 1.73, 1.51, *δ*_C_ 32.5), CH_2_-4 (*δ*_H_ 1.79, 1.50, *δ*_C_ 34.7), CH_2_-1 (*δ*_H_ 1.62, 1.45, *δ*_C_ 34.6), CH_2_-5 (*δ*_H_ 2.11, 1.77, *δ*_C_ 32.6), CH_2_-13 (*δ*_H_ 2.22, 2.11, *δ*_C_ 27.2); five sp^3^ methines including one oxygenated: CH-9 (*δ*_H_ 4.83, *δ*_C_ 70.0), CH-15 (*δ*_H_ 2.52, *δ*_C_ 27.0), CH-3 (*δ*_H_ 2.17, *δ*_C_ 41.5), CH-6 (*δ*_H_ 2.35, *δ*_C_ 45.7), and CH-2 (*δ*_H_ 1.71, *δ*_C_ 49.5); three sp^2^ quaternary carbon atoms: *δ*_C_ 134.4 (C-10), *δ*_C_ 150.8 (C-14), *δ*_C_ 153.9 (C-7), one sp^3^ quaternary carbon atom at *δ*_C_ 50.3 (C-11); and one carbonyl carbon atom at *δ*_C_ 203.5 (C-8). The presence of one carbonyl group and two pairs of double bonds, totaling three degrees of unsaturation, suggests that compound **4** is a diterpenoid featuring tricyclic systems.

**Fig. 5:**
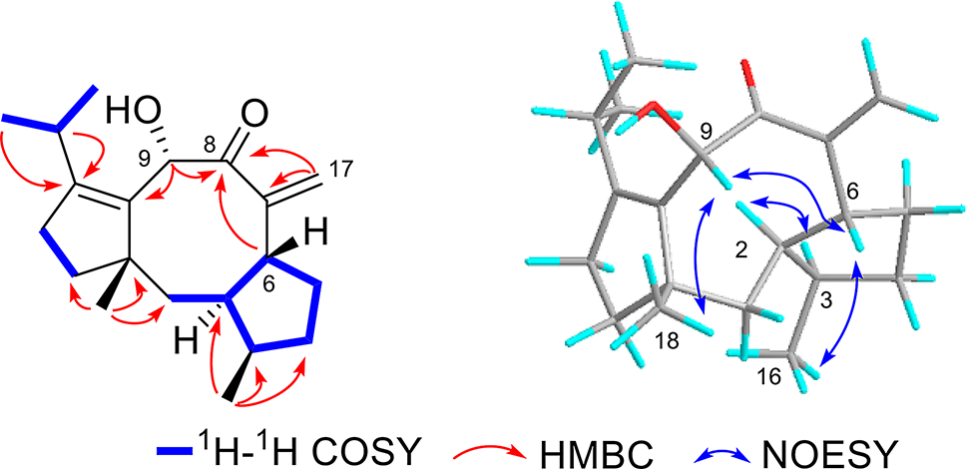
Selected 2D NMR correlations of **4**

The NMR data for compound **4** (Figs. S12–17) closely resembles that of compound **3** (Table 2). The primary distinction between compounds **3** and **4** lies in the absence of a hydroxyl group in **3** and the presence of a carbonyl group in **4**. The key HMBC (Fig. 5) correlations from H_2_-17, H-6, and H-9 to C-8, indicated the carbonyl group located in C-8. Biogenetically, the absolute configurations of all chiral carbon centers of **4** should be agreement well with those of **3**. In hence, the absolute structure of **4** was determined as shown in Fig. 1.

**Table 2:** ^1^H (600 MHz) and ^13^C NMR (150 MHz) data for **3**–**4**

NO.	3		4	
	δ_H_ (J in Hz)	δc	δ_H_ (J in Hz)	δc
1	1.59 m; 1.38, m	34.3, CH_2_	1.62 m; 1.45, m	34.6, CH_2_
2	1.66, m	50.3, CH	1.71, m	49.5, CH
3	2.03, m	41.4, CH	2.17, m	41.5, CH
4	1.68, m; 1.34, m	34.3, CH_2_	1.79, m; 1.50, m	34.7, CH_2_
5	2.13, m; 1.83, m	34.5, CH_2_	2.11, m; 1.77, m	32.6, CH_2_
6	1.89, m	45.8, CH	2.35, m	45.7, CH
7		155.7, C		153.9, C
8	4.50, dt (5.2, 2.2)	76.2, CH		203.5, C
9	4.37, d (5.2)	69.1, CH	4.83, d (2.2)	69.1, CH
10		137.9, C		134.4, C
11		50.5, C		50.3, C
12	1.55, m; 1.32, m	42.9, CH_2_	1.73, m; 1.51, m	44.1, CH_2_
13	2.17, m; 2.02, m	26.7, CH_2_	2.22, m; 2.11, m	27.2, CH_2_
14		146.8, C		150.8, C
15	3.16, sept (6.8)	28.3, CH	2.52, sept (6.8)	27.0, CH
16	0.79, d (7.1)	14.6, CH_3_	0.89, d (7.1)	14.9, CH_3_
17		106.5, CH_2_		120.0, CH_2_
18	0.98, s	27.2, CH_3_	1.12, s	27.2, CH_3_
19	0.97, d (6.7)	20.6, CH_3_	0.77, d (6.8)	19.2, CH_3_
20	0.94, d (7.0)	21.7, CH_3_	0.91, d (6.8)	21.0, CH_3_

### Cytotoxicity assay

**Fig. 6:**
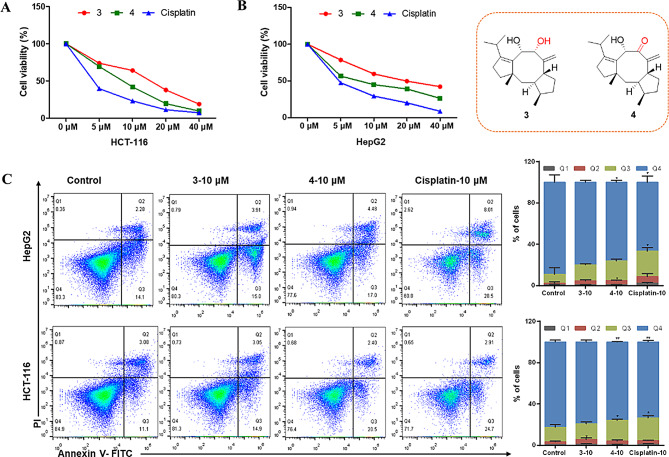
The cytotoxic activity of compounds **3** and **4**. **A**–**B**) Cell viability of HCT-116 and HepG2 cells treated by **3** or **4**. Cisplatin was used as the positive control. **C** Cell apoptosis of HCT-116 and HepG2 cells treated by **3** or **4**. Data were presented as the mean ± SEM (*n* = 3). *P < 0.05, **P < 0.01 vs. control

**Fig. 7:**
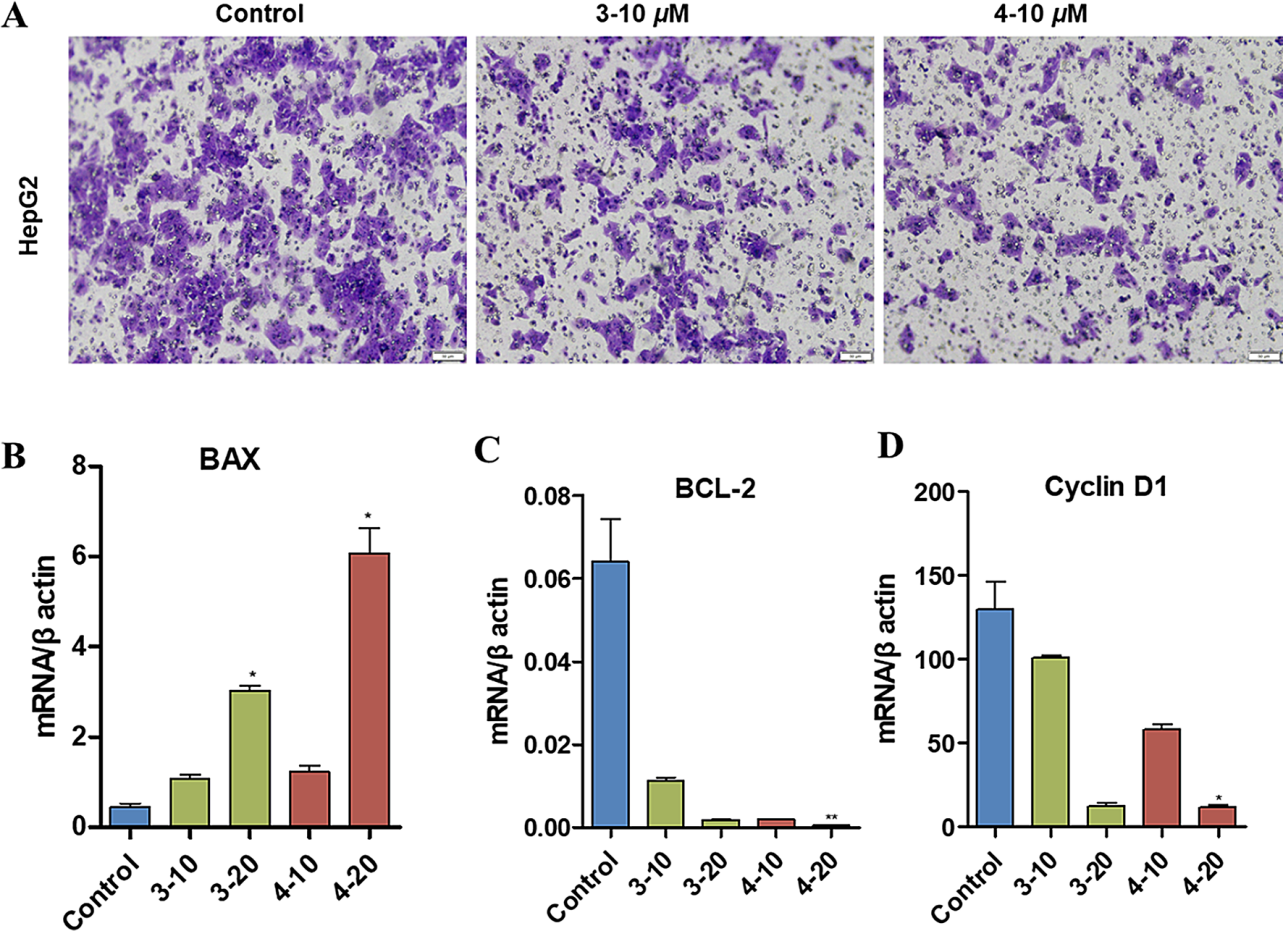
Compounds **3** and **4** inhibit cell migration and promote cell apoptosis of HepG2 cells. **A**) Cell migration of HepG2 cells treated by **3** or **4**. **B**–**D**) The transcription level of BAX, BCL-2 and cyclin D1 of HepG2 cells treated by **3** or **4**. Data were presented as the mean ± SEM (*n* = 3). *P < 0.05, **P < 0.01 vs. control

The cytotoxic activity of compounds **1**–**4** was evaluated against HCT-116 (colon cancer), A-549 (lung cancer), HepG2 (liver cancer), MCF-7 (breast cancer), SW1990 (pancreatic cancer). The cell survival assay was performed using the CCK-8 method (Xu et al. 2021). Each tumor cell line was exposed for 48 h to the tested compounds at concentrations ranging from 5 to 40 mM. **3** and **4** inhibit the cell viability of human cancer cell lines at the IC_50_ values ranging from 7.8 to 32.4 *µ*M (Table 3; Figs. 6A–B). Moreover, **3** and **4** also promote cell apoptosis of HepG2 cells and HCT-116 cells (Figs. 6C–D). Similarly, **3** and **4** suppress the immigration ability of HepG2 cells (Fig. 7A). As well, **3** and **4** also significantly decreased the gene level of BCL2 apoptosis regulator (BCL-2), and cyclin D1, while notably increased the transcription level BCL2 associated X, apoptosis regulator (BAX) (Figs. 7B–D).

**Table 3:** Inhibitory activity against human cancer cells of **1**–**4** (*µ*M)

Compound	HCT-116	A549	HepG2	MCF-7	SW1990
**1**	>40	>40	>40	>40	>40
**2**	>40	>40	>40	>40	>40
**3**	16.0 ± 1.2	16.6 ± 3.7	22.1 ± 1.3	32.4 ± 7.4	>40
**4**	8.3 ± 1.8	11.3 ± 1.1	7.8 ± 0.6	11.1 ± 1.5	20.1 ± 4.8
Cisplatin^a^	7.7 ± 0.9	8.6 ± 0.9	4. 5 ± 0.7	6.5 ± 0.8	7.5 ± 1.9

^a^Cisplatin was used as the positive control

## Concluding remarks

Focused on the topic in biosynthesis of sordarin, Tang (Sun et al. 2022) and Ge’s (Liu et al. 2022) research results differ from the speculated biosynthetic pathway by Fumitaka Kudo (Kudo et al. 2016), in that the B − V oxidation and Diels–Alder reaction cyclization are completed by four P450s working together to form a 5/5/6/5 tetracyclic stereochemical structure. They identified SdnB is used for C − C bond cleavage, resulting in the formation of a 5/5/6/5 tetracyclic stereochemical structure, which is not the role played by SdnN. Actually, the SdnG has been determined as a Diels-Alderase to catalyze the construction of complex 5/5/6/5 tetracyclic structure (Liu et al. 2022). However, compound **4** isolated by our experiment did not participate in this process. But they proposed that SdnB oxidizes the C-8/C-9 of **1** to form dihydroxyl groups (**3**), which is consistent with our results.

We obtained **1** by heterologous expression of *tdn*AC in *A. oryzae*. On this basis, we transformed four P450 genes *tdn*BEFH involved in oxidation to produce **2** − **4**. According to the research results, we speculate that TdnB oxidizes the C-8/C-9 position of **1** to form hydroxyl products **2** and **3**, and TdnB further oxidizes the C-8 position to form carbonyl compound **4** (Fig. 3C). The C-8 carbonyl group, due to its higher oxidation state, is the prerequisite for the next step of ring cleavage reaction and is also the key intermediate closest to the proposed biosynthetic pathway in the original literature.

Sordarin is a unique and promising fungal growth inhibitor. It binds to the fungal eEF2 and targets the main metabolic cycle, which has a unique active mechanism in antifungal activity, making it a better antibiotic than other antifungal compounds. Therefore, the application of sordarins should be expanded from a useful tool for studying eukaryotic translation systems to clinical treatment of fungal infections. In order to improve the application of sordarin as a clinical antibiotic, a low-cost and efficient industrial production method needs to be developed. The full analysis of the biosynthetic pathway of sordarins laying a theoretical foundation for the industrial production of sordarins.

In summary, we found a new BGC from *T. adpressus*, and a new intermediate **4** in the biosynthetic pathway of sordarins using genome mining method. Moreover, all isolates were evaluated for their bioactivities, and compounds **3** and **4** showed cytotoxic activity against human cancer cell lines HCT116, A549, HepG2, MCF-7 and SW1990. Among which, **4** exerts superior anti-tumor ability on cell apoptosis and cell migration. Targets predication and molecular docking indicate that compound **4** exhibits stronger affinity for DBL, suggesting its excellent binding potential (Figs. S18 and S19). It prompts that **4** suppress cancer cell proliferation and migration by targeting beta-PIX. This groundbreaking finding will offer new strategies for synthetic biological research into sordarins and significantly enrich the structures and bioactivities of natural diterpenoids featuring a tricyclic 5/8/5 ring system, marking a significant advancement in the field.

## Electronic supplementary material

Below is the link to the electronic supplementary material.

Supplementary Material 1

Supplementary Material 2

Supplementary Material 3

Supplementary Material 4

## Data Availability

The data supporting the conclusions are included in the main manuscript. And the HRESIMS, 1D and 2D NMR, UV, and IR spectra of **3**–**4**, and the 1D NMR of **1**–**2** can be found online at https://bioresourcesbioprocessing.springeropen.com.
