# Supplementary material for: Uncovering a novel biosynthetic gene cluster for sordarin through genome mining in the fungus Talaromyces adpressus
Source: Bioresour Bioprocess. 2025 Apr 17;12(1):35. doi: 10.1186/s40643-025-00864-x (PMC12006653; doi:10.1186/s40643-025-00864-x)

**Supporting information**

Uncovering a Novel Biosynthetic Gene Cluster for Sordarin through Genome Mining in the Fungus *Talaromyces adpressus*

Qianqian Xu,^1,3,^† Xiaomeng Ren,^3,4,^† Linzhen Hu,^2,^† Qiaoxin Xu,^3^ Xiaodong Zhang,^2^ Mengyi Deng,^5^ Ying Ye,^3,^* Yonghui Zhang,^3,^* Yuanyuan Lu,^1,^* Yuben Qiao,^2,3,^*

^1^ Maternal and Child Health Hospital of Hubei Province, Tongji Medical College, Huazhong University of Science and Technology, Wuhan 430033, People’s Republic of China

^2^ State Key Laboratory of Biocatalysis and Enzyme Engineering, School of Life Sciences, Hubei University, Wuhan 430062, People’s Republic of China

^3^ Hubei Key Laboratory of Natural Medicinal Chemistry and Resource Evaluation, School of Pharmacy, Tongji Medical College, Huazhong University of Science and Technology, Wuhan 430030, People’s Republic of China

^4^ Innovation Laboratory for Sciences and Technologies of Energy Materials of Fujian Province (IKKEM), Xiamen 361005, People’s Republic of China

^5^ School of Life Science and Engineering, Southwest Jiaotong University, Chengdu, 610031, Sichuan, People’s Republic of China

* Corresponding authors.

*E-mail addresses*: qiaoyuben12@163.com (Y. Qiao); luyuanyuan200715@163.com (Y. Lu); zhangyh@mails.tjmu.edu.cn (Y. Zhang); ying_ye@hust.edu.cn (Y. Ye).

**Content**

[Table S1. Oligonucleotides used for construction of plasmids. 3](#_Toc187909503)

[Table S2. Summary of the transformants constructed in this study. 3](#_Toc187909504)

[Table S3. A list of primers used in real-time PCR analysis. 3](#_Toc187909505)

[Table S4. The potential targets of compound **4** predicted by PharmMapper database. 1](#_Toc187909506)

[Fig. S1. ^1^H NMR (400 MHz) spectrum of compound **1** (Recorded in CH_3_Cl-*d*). 2](#_Toc187909507)

[Fig. S2. ^13^C NMR (100 MHz) spectrum of compound **1** (Recorded in CH_3_Cl-*d*). 3](#_Toc187909508)

[Fig. S3. ^13^C NMR (150 MHz) spectrum of compound **2** (Recorded in CH_3_Cl-*d*). 4](#_Toc187909509)

[Fig. S4. HRESIMS spectra of compound **3** (A) and **4** (B). 5](#_Toc187909510)

[Fig. S5. IR and UV spectra of compound **3** (A and B) and **4** (C and D). 6](#_Toc187909511)

[Fig. S6. ^1^H NMR (600 MHz) spectrum of compound **3** (Recorded in CH_3_Cl-*d*). 7](#_Toc187909512)

[Fig. S7. ^13^C NMR (150 MHz) spectrum of compound **3** (Recorded in CH_3_Cl-*d*). 8](#_Toc187909513)

[Fig. S8. HSQC spectrum of compound **3** (Recorded in CH_3_Cl-*d*). 9](#_Toc187909514)

[Fig. S9. HMBC spectrum of compound **3** (Recorded in CH_3_Cl-*d*). 10](#_Toc187909515)

[Fig. S10. ^1^H–^1^H COSY spectrum of compound **3** (Recorded in CH_3_Cl-*d*). 11](#_Toc187909516)

[Fig. S11. NOESY spectrum of compound **3** (Recorded in CH_3_Cl-*d*). 12](#_Toc187909517)

[Fig. S12. ^1^H NMR (600 MHz) spectrum of **4** (Recorded in CH_3_Cl-*d*). 13](#_Toc187909518)

[Fig. S13. ^13^C NMR (150 MHz) spectrum of compound **4** (Recorded in CH_3_Cl-*d*). 14](#_Toc187909519)

[Fig. S14. HSQC spectrum of compound **4** (Recorded in CH_3_Cl-*d*). 15](#_Toc187909520)

[Fig. S15. HMBC spectrum of compound **4** (Recorded in CH_3_Cl-*d*). 16](#_Toc187909521)

[Fig. S16. ^1^H–^1^H COSY spectrum of compound **4** (Recorded in CH_3_Cl-*d*). 17](#_Toc187909522)

[Fig. S17. NOESY spectrum of compound **4** (Recorded in CH_3_Cl-*d*). 18](#_Toc187909523)

[Targets prediction and molecular docking 19](#_Toc187909524)

[Fig. S18. Molecular docking for compounds **1**–**4**. 20](#_Toc187909525)

[Fig. S19. Zoomed-in view of the DBL binding site in complex with compound **4**. 21](#_Toc187909526)

# **Table S1**. Oligonucleotides used for construction of plasmids.

| **Amplicon** | **Sequence (from 5’ to 3’)** |
| --- | --- |
|  |  |
| *pAdeA2-tdnA-F/R* | CGAATTCGAATCGATTTGAGCTAGCATGTCACTGTACGGTTTATTTT |
|  | CCGGGTCACTAGTGCGGCCGCTAGCCTATGGAAGATCCAAGGTTCTT |
| *pUARA2-tdnB-F/R* | CCGGAATTCGAGCTCGGTACCATGGCATATCCCTCGCCCAC |
|  | AGCTACTACAGATCCCCGGGTACCCTATGTAAGAACAGACGTGACCC |
| linker | GGTACCCGGGGATCTGTAGT |
|  | GCTAGCTCAAATCGATTCGA |
| *pAdeA2-TdnC-F/R* | CAGCAAGCTCCGGAATTCGAGCTCGGTACCATGGACAACAAGACAGCAC |
|  | GAGCTACTACAGATCCCCGGGTACCCTATACTTTCAAAACCTCAAAC |
| *pUARA2-tdnE-F/R* | ATTCGAATCGATTTGAGCTAGCATGGAGTTCTATACCGCATCTT |
|  | CCGGGTCACTAGTGCGGCCGCTAGTCACTTGTTGTAAGCTGCTTTT |
| *pUARA2-tdnF-F/R* | AGCTCCGGAATTCGAGCTCGGTACCATGGCTTCGCTCGAGTGCCC |
|  | TACTACAGATCCCCGGGTACCCTACGCTGCGAGGTCGAGTG |
| *pUARA2-tdnH-F/R* | AATTCGAATCGATTTGAGCTAGCATGTTGGACCTTGGAGAACCA |
|  | GGGTCACTAGTGCGGCCGCTAGTTAGACGACGAAAGCATTAAGA |

# **Table S2**. Summary of the transformants constructed in this study.

| Transformants | **Plasmids** (selection markers) | | |
| --- | --- | --- | --- |
|  | *AdeA* | *ArgB* | *sC* |
| AO*-tdnAC* | pAdeA2*-tbnAC* | *-* | *-* |
| AO*-tdnACBEFH* | pAdeA2*-tdnAC* | pUARA2*-tdnBEFH* | *-* |

# **Table S3**. A list of primers used in real-time PCR analysis.

| human gene | Sense | Anti sense |
| --- | --- | --- |
| Bcl-2 | GACTTCTCCCGCCGCTACCG | CCCAGTTCACCCCGTCCCT |
| Bax | TTTCTGACGGCAACTTCAACTGG | TGATCAGTTCCGGCACCTT |
| Cyclin D1 | GCCCAGCAGAACATGGACCC | GCCCTCAGATGTCCACGTCCC |
| β-actin | CGTGCGTGACATCAAAGAGAA | TGGATGCCACAGGATTCCAT |

# **Table S4**. The potential targets of compound 4 predicted by PharmMapper database.

| No. | Pharma Model | Fit | Norm Fit | Num Hydrophobic | Name | Uniplot | Function |
| --- | --- | --- | --- | --- | --- | --- | --- |
| 1 | 2ziw_A_cavity_1 | 2.948 | 0.9826 | 3 | Crossover junction endonuclease MUS81 | Q7SXA9 | NONE |
| 2 | 3wxv_A_cavity_5 | 2.937 | 0.979 | 3 | NONE | NONE | NONE |
| 3 | 1i0s_A_cavity_2 | 2.901 | 0.9669 | 3 | Hypothetical protein | O29428 | Involved in oxidoreductase activity |
| 4 | 1by1_A_cavity_1 | 2.895 | 0.965 | 3 | Rho guanine nucleotide exchange factor 7 | ARHG7_HUMAN | Acts as a RAC1 guanine nucleotide exchange factor (GEF) and can induce membrane ruffling (By similarity). |
| 5 | 1igw_B_cavity_3 | 2.889 | 0.9629 | 3 | Isocitrate lyase | P0A9G6 | NONE |
| 6 | 3f5c_B_cavity_1 | 2.881 | 0.9604 | 3 | Nuclear receptor subfamily 5 group A member 2 | P45448 | NONE |
| 7 | 1e0y_B_cavity_6 | 2.878 | 0.9593 | 3 | Vanillyl-alcohol oxidase | P56216 | Energy production and conversion |
| 8 | 2nq2_B_cavity_1 | 2.871 | 0.9569 | 3 | Probable ABC transporter permease protein HI1471 | Q57130 | NONE |
| 9 | 1pjr_A_cavity_5 | 2.867 | 0.9555 | 3 | ATP-dependent DNA helicase pcrA | P56255 | NONE |
| 10 | 3hzq_A_cavity_2 | 2.861 | 0.9536 | 3 | Large-conductance mechanosensitive channel | P68806 | NONE |

# **Fig. S1.** ^1^H NMR (400 MHz) spectrum of compound 1 (Recorded in CH_3_Cl-*d*).

# **Fig. S2.** ^13^C NMR (100 MHz) spectrum of compound 1 (Recorded in CH_3_Cl-*d*).

# **Fig. S3.** ^13^C NMR (150 MHz) spectrum of compound 2 (Recorded in CH_3_Cl-*d*).

# **Fig. S4.** HRESIMS spectra of compound 3 (A) and 4 (B).


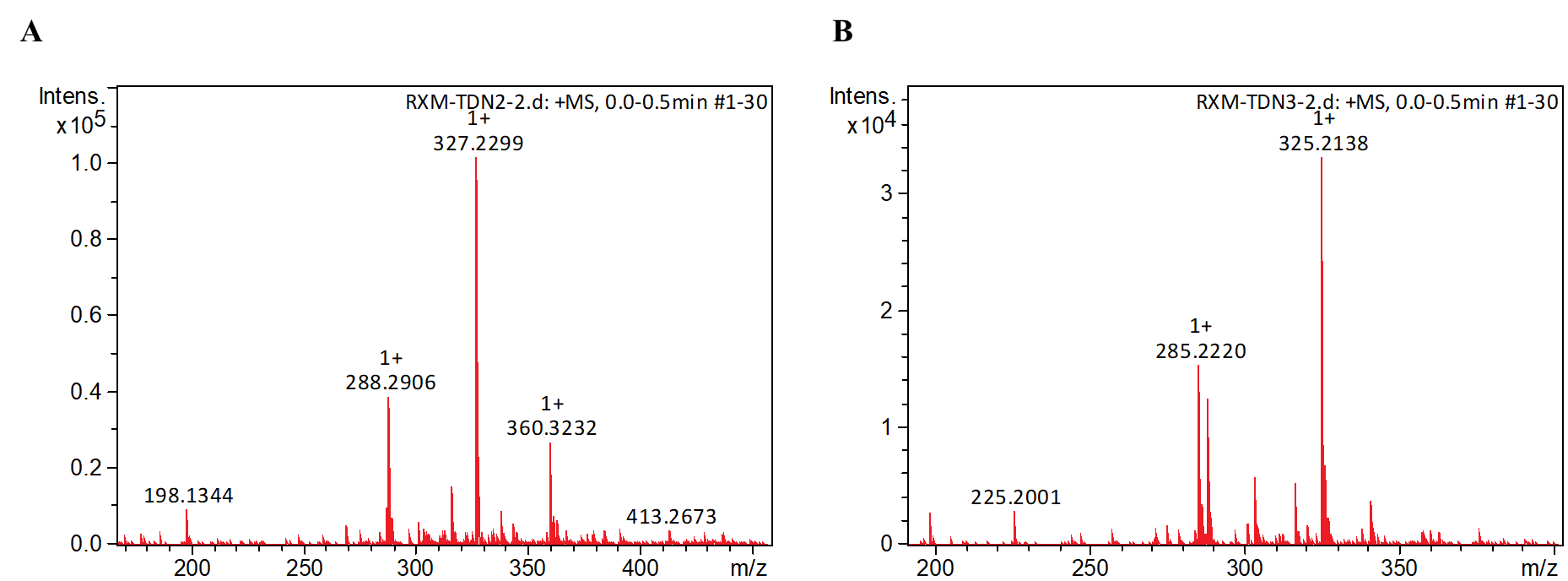


# **Fig. S5.** IR and UV spectra of compound 3 (A and B) and 4 (C and D).


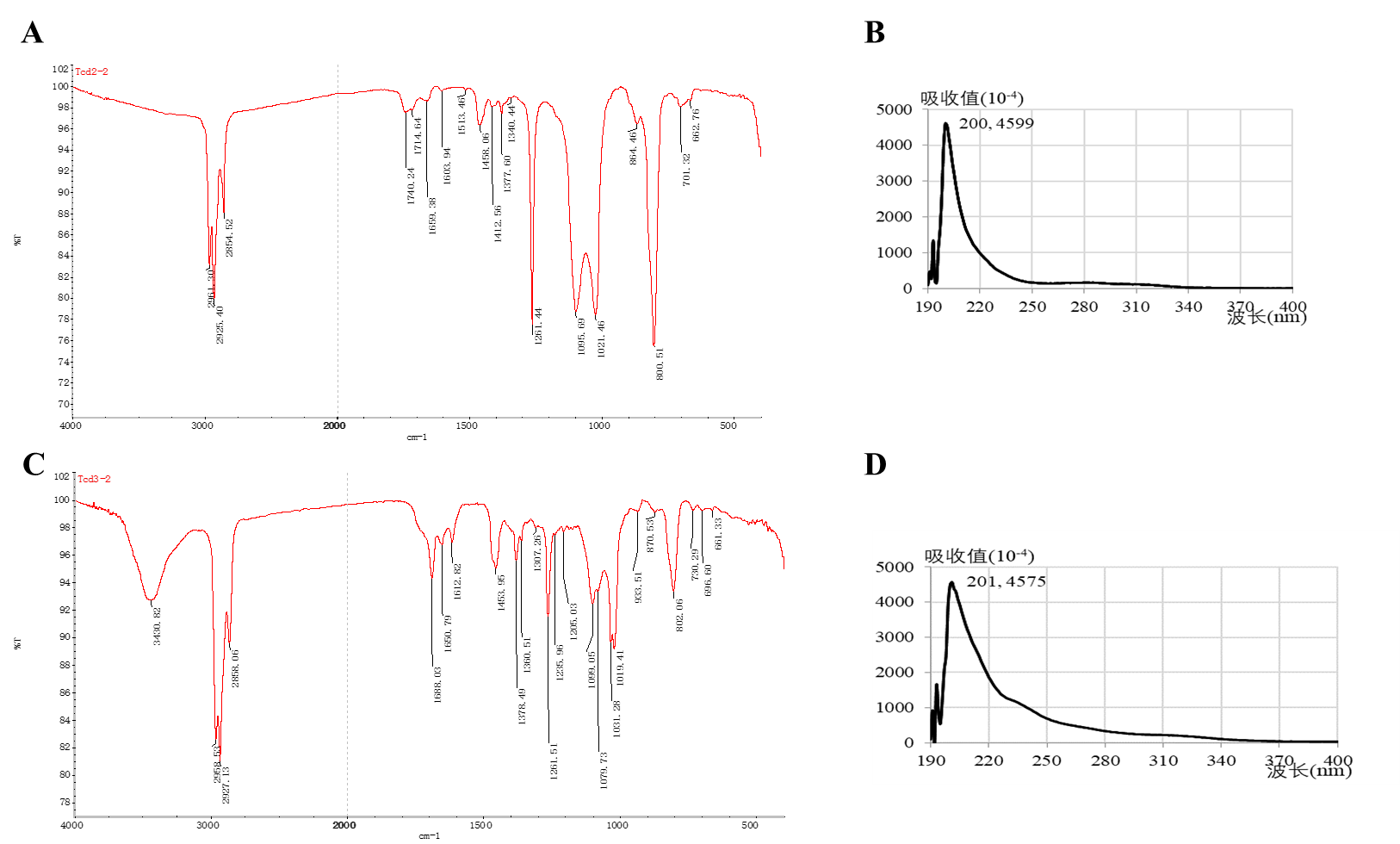


# **Fig. S6.** ^1^H NMR (600 MHz) spectrum of compound 3 (Recorded in CH_3_Cl-*d*).

# **Fig. S7.** ^13^C NMR (150 MHz) spectrum of compound 3 (Recorded in CH_3_Cl-*d*).

# **Fig. S8.** HSQC spectrum of compound 3 (Recorded in CH_3_Cl-*d*).

# **Fig. S9.** HMBC spectrum of compound 3 (Recorded in CH_3_Cl-*d*).

# **Fig. S10.** ^1^H–^1^H COSY spectrum of compound 3 (Recorded in CH_3_Cl-*d*).

# **Fig. S11.** NOESY spectrum of compound 3 (Recorded in CH_3_Cl-*d*).

# **Fig. S12.** ^1^H NMR (600 MHz) spectrum of 4 (Recorded in CH_3_Cl-*d*).

# **Fig. S13.** ^13^C NMR (150 MHz) spectrum of compound 4 (Recorded in CH_3_Cl-*d*).

# **Fig. S14.** HSQC spectrum of compound 4 (Recorded in CH_3_Cl-*d*).

# **Fig. S15.** HMBC spectrum of compound 4 (Recorded in CH_3_Cl-*d*).

# **Fig. S16.** ^1^H–^1^H COSY spectrum of compound 4 (Recorded in CH_3_Cl-*d*).

# **Fig. S17.** NOESY spectrum of compound 4 (Recorded in CH_3_Cl-*d*).

**Targets prediction and molecular docking**

To explore the mechanism of these compounds in exerting significant anti-tumor effects, the potential targets of **1**–**4** was predicted using the PharmMapper database (Wang et al., 2017), the ten most potential targets were listed in Table S3. Among which, the beta-PIX (also known as ARHG7) protein was reported to participate in the invasion and migration of multiple malignancies (Lei et al., 2018; Li et al., 2002; Yu et al., 2015). Hence, we explored the binding ability of compounds **1**–**4** to the DBL homology domain from beta-PIX (PDB: 1BY1). The docking results revealed that all the compounds primarily bound to the loop region at the N-terminal of the DBL protein. The binding energies for **1**–**4** was calculated as 6.164 kcal/mol, 6.115 kcal/mol, 6.185 kcal/mol, and 6.581 kcal/mol (more positive energies indicate stronger binding), respectively (Figure S18). Notably, compound **4** exhibited the highest binding energy, attributed to an additional keto group that formed a stable hydrogen bond with the amino group of the Y14 backbone, further stabilizing its interaction with the protein (Figure S19). These findings indicate that compound **4** exhibits anti-tumor activity by targeting beta-PIX.

Lei, X., Deng, L., Liu, D., Liao, S., Dai, H., Li, J., Rong, J., Wang, Z., Huang, G., Tang, C., Xu, C., Xiao, B., Li, T. 2018. ARHGEF7 promotes metastasis of colorectal adenocarcinoma by regulating the motility of cancer cells. *Int J Oncol*, **53**(5), 1980-1996.

Li, Z.-y., Wang, Y.-j., Song, J.-p., Kataoka, H., Yoshii, S., Gao, C.-m., Wang, Y.-p., Zhou, J.-n., Ota, S., Tanaka, M., Sugimura, H. 2002. Genomic structure of the human β-PIX gene and its alteration in gastric cancer. *Cancer Letters*, **177**(2), 203-208.

Wang, X., Shen, Y., Wang, S., Li, S., Zhang, W., Liu, X., Lai, L., Pei, J., Li, H. 2017. PharmMapper 2017 update: a web server for potential drug target identification with a comprehensive target pharmacophore database. *Nucleic Acids Res*, **45**(W1), W356-w360.

Yu, H.W., Chen, Y.Q., Huang, C.M., Liu, C.Y., Chiou, A., Wang, Y.K., Tang, M.J., Kuo, J.C. 2015. β-PIX controls intracellular viscoelasticity to regulate lung cancer cell migration. *J Cell Mol Med*, **19**(5), 934-47.

# **Fig. S18.** Molecular docking for compounds 1–4.


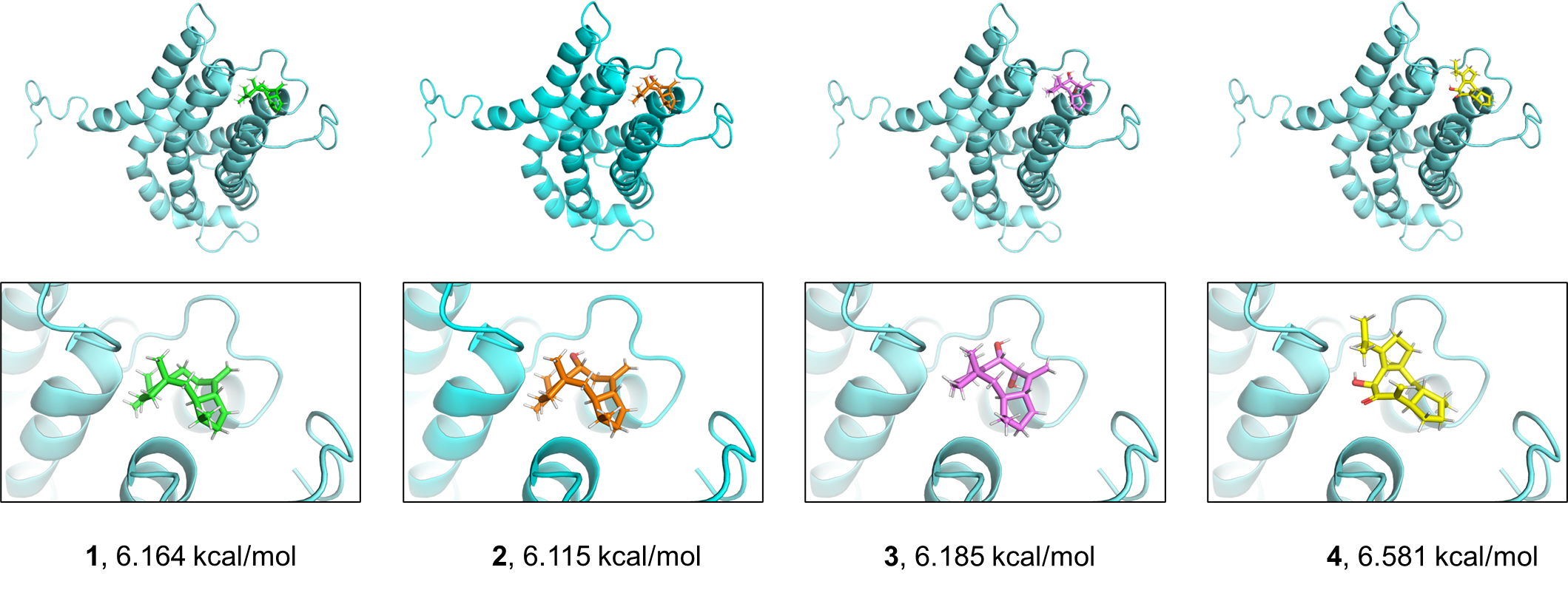


# **Fig. S19.** Zoomed-in view of the DBL binding site in complex with compound 4.


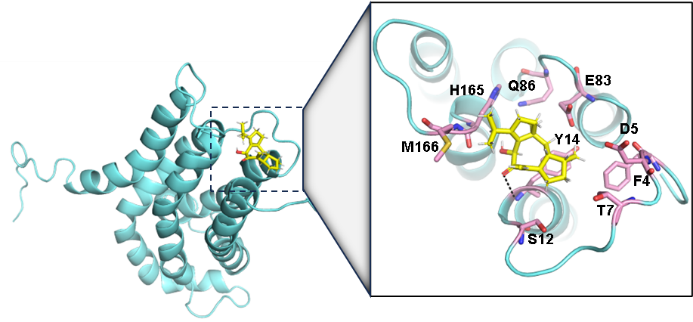

Supplement: Supplementary file 1 — Supplementary Material 1 [file 40643_2025_864_MOESM1_ESM.docx]
