# Supplementary material for: Uncovering a novel biosynthetic gene cluster for sordarin through genome mining in the fungus Talaromyces adpressus
Source: Bioresour Bioprocess. 2025 Apr 17;12(1):35. doi: 10.1186/s40643-025-00864-x (PMC12006653; doi:10.1186/s40643-025-00864-x)
Supplement: Supplementary file 2 — Supplementary Material 2 [file 40643_2025_864_MOESM2_ESM.doc]

**Lists of Response**

**Dear Reviewer 1:**

Thanks a lot for your nice suggestions concerning the manuscript. We believe that these comments would improve the manuscript with high quality. In manuscript-R2, the corresponding revisions have been marked in yellow in text. We hope it will meet your approval. The responses to all the comments have been listed below.

**Comment 1**: Regarding whether the gene cluster is a sordarin biosynthetic gene cluster, the authors supplemented the results of mass spectrometry detection showing that Aspergillus oryzae with the entire gene cluster tdnACBEFH produced sordaricin. However, why was the extracted molecular weight 347.2222 instead of the precise molecular weight of the compound, 347.3154? Moreover, why was the TIC (Total Ion Current) plot used for the wild-type Aspergillus oryzae instead of extracting ion chromatograms?

**Response**: We sincerely appreciate your insightful feedback regarding the methodological inconsistencies in our previous analysis. In response to your valuable suggestions, we have conducted a comprehensive reanalysis of the mass spectrometry data to accurately determine the molecular weight differences between the AO-tdnACBEFH engineered strain and wild-type *Aspergillus oryzae*.

The revised results (Figure as follows) incorporate these critical corrections, with the previous version retained to facilitate comparative assessment. This recalibration has significantly improved the precision of our compound characterization. We are grateful for your rigorous review that has substantially strengthened the scientific validity of this study. Please find attached the updated figure.


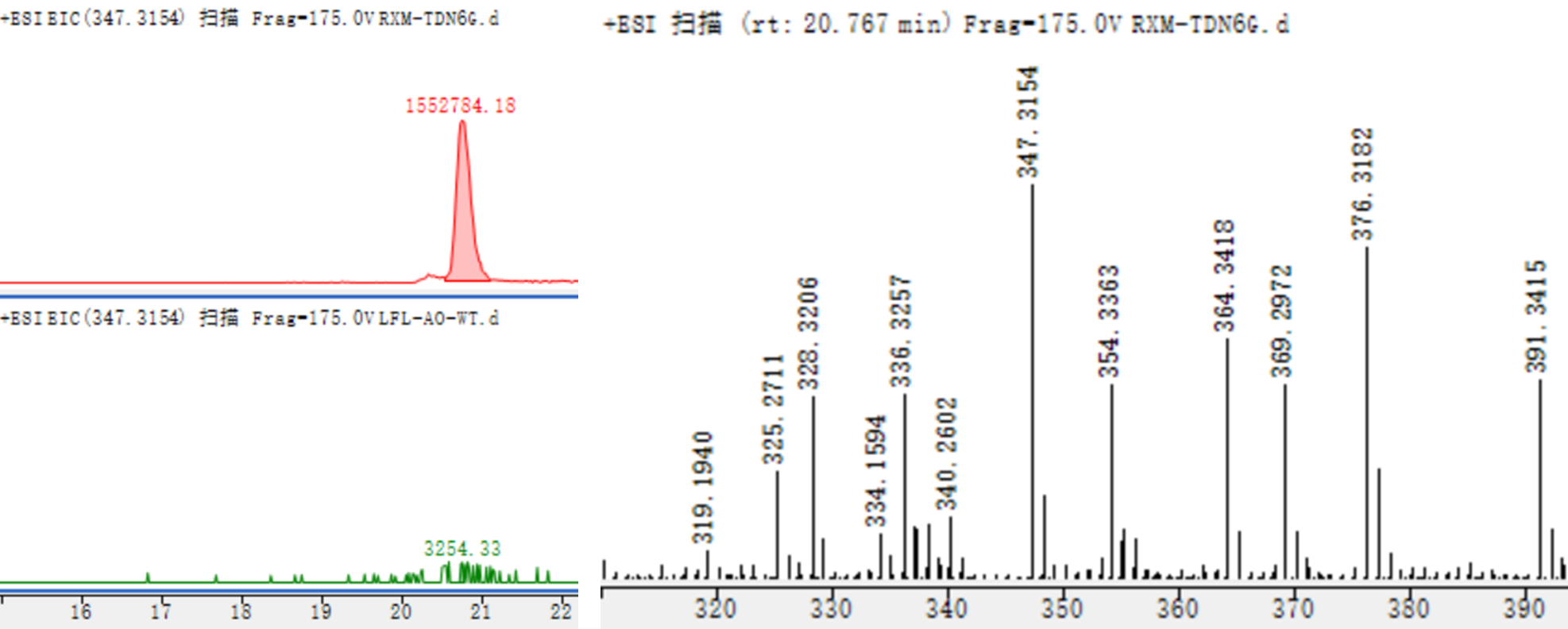


New figure for review


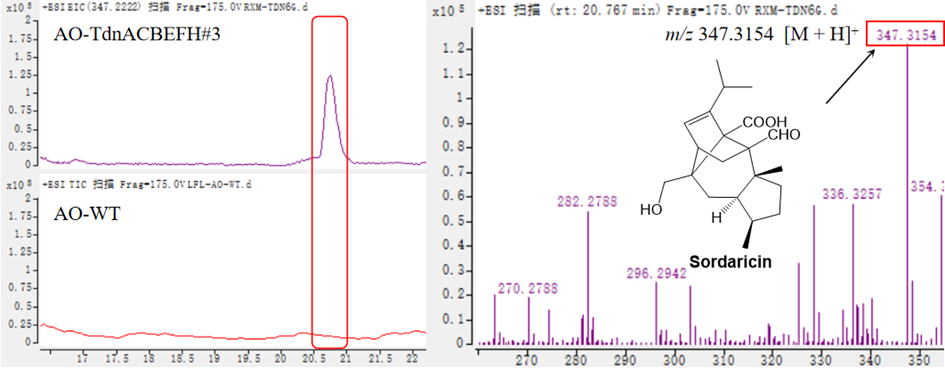


Previous figure for review

Reviewer 1#comment 2: Without additional experimental evidence, it is not possible to conclude that TdnB catalyzes multi-step oxidation to form compounds 2 and 3, especially the new compound 4, as stated in the second paragraph of the concluding remarks and Figure 3C. Additionally, Figure 3D should be corrected to Figure 3C. Furthermore, what is the adjusted figure in Figure 4A? This should be clarified in the figure legend.

**Response**: We sincerely appreciate your expert guidance on improving the mechanistic interpretation. As demonstrated in the seminal work by Tang and Ge, the cytochrome P450 enzyme in the *sdn* gene cluster mediates complete substrate oxidation including C-C bond cleavage and skeletal rearrangement, ultimately generating novel products. Significantly, our phylogenetic analysis reveals high sequence homology (Table 1) between the *tdn*-encoded P450s and *sdn* counterparts, strongly supporting the potential conservation of this oxidative cascade in our system.

Additionally, in the Baeyer-Villiger oxidation pathway, the characteristic cleavage of the C8-C9 bond in compound **4** may represent a critical intermediate transformation. While the structural congruence between this product and established biosynthetic intermediates suggests potential biological relevance, our current investigation strategically focuses on the structural elucidation and pharmacological characterization of these new natural products rather than definitive biosynthetic pathway elucidation.

Per your recommendations, we have revised Figure 3D as the Figure 3C. Annotated the RT20-min peaks (*) in Figure 4A with the following legend clarification: "*Signals corresponding to non-target compounds (RT 19.8±0.2 min) excluded from structural series **1**-**4** based on MS/MS fragmentation patterns".

**Dear Reviewer 2:**

Thanks a lot for your nice suggestions concerning the manuscript. We believe that these comments would improve the manuscript with high quality. In manuscript-R2, the corresponding revisions have been marked in yellow in text. We hope it will meet your approval. The responses to all the comments have been listed below.

**Comment 1:** The previous studies should be mentioned in the introduction part. Also, Table 1 should be updated based on the current understanding of the biosynthesis.

**Response**: Thank you very much for your advice. According to your suggestions, we have added the previous research to the Introduction part, which is marked in yellow. Please see the new vision. Many thanks for your advices again.

Response #2: If the authors wish to include tdnX, it should be included in Table 1 and briefly mentioned in the main text.

**Response**: Thank you very much for your advice which has been very helpful in improving our manuscript. According to previous research, the sdnX would not be involving in the biosynthesis of sordarin. However, in order to enable readers to obtain as much information as possible, we have also placed it in table 1. Due to the uncertainty of the function of tdnX, we did not elaborate more on it in the main text. Thanks for your advices again.

Response #3: There are no comments or additional experiments regarding the intermediacy of compound 4.

**Response**: We sincerely appreciate your rigorous evaluation and valuable professional feedback. As indicated in our previous responses and supported by existing biosynthetic literatures, we have tentatively proposed that the highly oxidized structure of compound **4** may represent a biosynthetic intermediate. However, it should be emphasized that the elucidation of biosynthetic pathways falls beyond the current scope of our investigation. Given that our primary research focus remains on the discovery and functional characterization of new chemical entities, we have deliberately prioritized the structural identification and bioactivity profiling of this new compound over mechanistic validation of its potential intermediary role in biosynthetic processes. The present study specifically aims to establish compound **4**'s chemical novelty and evaluate its biological properties, which we believe constitutes the most scientifically pertinent contribution of this work.
