# Supplementary figures and images for: Uncovering a novel biosynthetic gene cluster for sordarin through genome mining in the fungus Talaromyces adpressus
Source: Bioresour Bioprocess. 2025 Apr 17;12(1):35. doi: 10.1186/s40643-025-00864-x (PMC12006653; doi:10.1186/s40643-025-00864-x)

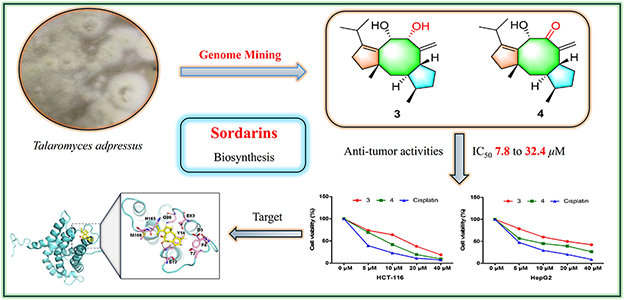

Supplement: Supplementary file 4 — Supplementary Material 4 [file 40643_2025_864_MOESM4_ESM.jpg]
