# Supplementary material for: Uncovering a novel biosynthetic gene cluster for sordarin through genome mining in the fungus Talaromyces adpressus
Source: Bioresour Bioprocess. 2025 Apr 17;12(1):35. doi: 10.1186/s40643-025-00864-x (PMC12006653; doi:10.1186/s40643-025-00864-x)
Supplement: Supplementary file 3 — Supplementary Material 3 [file 40643_2025_864_MOESM3_ESM.doc]

Yuben Qiao

State Key Laboratory of Biocatalysis and Enzyme Engineering

School of Life Sciences, Hubei University

368 Youyi Road, Wuhan 430062, CHINA

qiaoyuben12@163.com

Feb. 14^th^, 2025

Dear Prof. Jian-He Xu,

Thank you very much for giving us the opportunity to revise our manuscript (ID: BIOB-D-24-00302R1). According to the reviewer's comments, we have made great improvements in the new version. The corresponding revisions have been conducted point-by-point and marked in yellow in Manuscript-R2. Please accept our sincere gratitude for your rigorous and careful review on the manuscript.

We would like to express our great appreciation to you and the reviewers for all comments on our paper and hope the present revision is acceptable.

Looking forward to hearing from you soon.

With best regards,

Yuben Qiao
